# Eye movement and pupillary response abnormalities measured using virtual reality as biomarkers in the diagnosis of early-stage Parkinson’s disease

**DOI:** 10.3389/fneur.2025.1537841

**Published:** 2025-04-23

**Authors:** Jing Zhao, Chong Shi, Xucheng Zhang, Shaochen Ma, Wei Sun, Feng Tian, Peifu Wang, Jilai Li, Jichen Du, Xingquan Zhao, Zhirong Wan

**Affiliations:** ^1^Department of Neurology, Aerospace Center Hospital, Beijing, China; ^2^Department of Traditional Chinese Medicine, Aerospace Center Hospital, Beijing, China; ^3^Institute of Software, Chinese Academy of Sciences, Beijing, China; ^4^School of Artificial Intelligence, University of Chinese Academy of Sciences, Beijing, China; ^5^Department of Neurology, Beijing Tiantan Hospital, Capital Medical University, Beijing, China

**Keywords:** Parkinson’s disease, eye movement, pupillary response, virtual reality, diagnosis

## Abstract

**Objective:**

Characteristic ocular symptoms are expected to serve as potential biomarkers for early diagnosis of Parkinson’s disease (PD). However, possible ocular impairments in PD patients are rarely studied. The study aimed to investigate eye movement characteristics and pupil diameter changes in early-stage PD patients using virtual reality (VR)-based system and explore their contribution in the diagnosis of early-stage PD.

**Methods:**

Forty-three early-stage PD patients and 25 healthy controls were included. Eye movements and pupillary response of all subjects were recorded and evaluated by wearing VR glasses. All subjects completed pro-saccade and anti-saccade tasks. Saccadic eye movement and pupillary response parameters were analyzed. Random Forests method was used for classification task, the performance of the classification model in differentiating early-stage PD patients from healthy controls were evaluated.

**Results:**

PD patients exhibited reduced pro-saccade velocity and accuracy, longer average time to complete the pro-saccade, and lower anti-saccade error correction rate than healthy controls (all *p* < 0.05). Significant differences were found in the trajectories of changes in pupil diameter between the two groups. After extraction of frequency-amplitude features of pupil constriction from the spectra of the eye movement signals of PD patients, it can be seen that the amplitudes of movement signals of both the left and right eyes at different frequencies during pro-saccade and anti-saccade tasks were significant. The number of significant amplitude frequencies in both eyes at low (0–6 Hz), medium (7–12 Hz) and high frequencies (13–19 Hz) was 23, 9, and 16, respectively, during pro-saccade task, which was 10, 29, and 43, respectively, during anti-saccade task. The model with all features achieved an accuracy of up to 79%.

**Conclusion:**

This study presents a non-invasive approach toward the diagnosis of early-stage PD with VR technology. Eye movement and pupillary response abnormalities measured using VR may be used as effective biomarkers for the diagnosis of early-stage PD.

## 1 Introduction

Parkinson’s disease (PD) is a common neurodegenerative disease of middle-aged and older people, with degeneration and death of dopaminergic neurons in the nigrostriatal system as the main pathological changes. Clinically, PD is characterized by movement disorders, such as bradykinesia, resting tremor and rigidity. PD patients can also experience non-motor symptoms such as sensory impairments and autonomic dysfunction (1). Among non-motor symptoms, ocular symptoms and signs such as blurry vision, reading difficulty are often overlooked, which adversely affect the daily life of PD patients and increase caregiver burden (2). Ocular symptoms have gained greater attention in recent years. Since eye movements are closely related to structures such as the basal ganglia and brainstem, an increasing number of studies (3, 4) have demonstrated abnormalities in the visual pathway in PD patients.

Ocular symptoms in PD patients mainly include eye movement disorders and pupillary response abnormalities. Since dysfunction in neurological disorders often precedes imaging abnormalities, ocular symptoms are of great value in the early diagnosis of PD. Currently, there are only a few adjunctive or remote methods available to help clinicians to assess ocular symptoms consistently, which are highly sensitive objective measures. Saccade is a widely used cognitive test that is widely used in the detection of various neurodegenerative diseases (5, 6). A previous study showed that a specific type of eye movement had a sensitivity of 87% and specificity of 96% for the diagnosis of early-stage PD. (7) For studies using pupil constriction, existing research has achieved an 89% recognition rate for PD people using frequency analysis methods (8). Characteristic ocular symptoms are expected to serve as potential biomarkers for early diagnosis of PD. However, to date, there has been relatively limited research on investigating possible ocular impairments in PD patients, and no definitive conclusions have been drawn.

The purpose of this study was to investigate the characteristics of eye movements and pupillary response in patients with early-stage PD using virtual reality (VR)-based system, hoping to provide a promising, non-invasive and objective method for detecting biomarkers used to diagnose early-stage PD.

## 2 Methods

### 2.1 Participants

Forty-three patients with early-stage PD who attended the Parkinson’s Specialized Disease Clinic of the Aerospace Center Hospital from August 2023 to January 2024 were included. During the same period, 25 healthy volunteers were recruited as controls.

All subjects underwent neurological examination and diagnosed by two professionally trained neurologists specializing in the diagnosis and treatment of PD.

The inclusion criteria for PD patients were as follows: a clear diagnosis of PD based on the clinical diagnostic criteria for Parkinson’s disease introduced by the Movement Disorder Society in 2015 (9), with Hoehn and Yahr (H&Y) stages 1–2.5 (10). The exclusion criteria were as follows: age >75 years old; comorbidity with acute neurological diseases, the presence of severe visual impairment, obvious eye movement abnormalities or red-green color blindness, obvious head tremor and a previous history of seizures and epilepsy; the presence of hearing impairment, accompanied by severe cognitive impairment [Mini-Mental State Examination (MMSE) score <24 an and an MMSE score of <20 for those with an education level of elementary school or below] and diseases affecting videonystagmography tests; patients who underwent deep brain stimulation.

Subjects in the healthy control group had no history of neurological diseases, with no positive signs being found on neurological examination.

The study was approved by the Ethics Committee of the Aerospace Center Hospital (approval number: 2021-ASCH-009). Written informed consent was obtained from all subjects included in this study.

### 2.2 Clinical data collection

The baseline data of all subjects were collected, including age, sex, age of onset, duration of PD and prior medical history. Each participant completed a series of saccade and anti-saccade tasks during the data collection process, with each task consisting of 10 trials. Prior to the commencement of the testing, the instrument underwent calibration. Following the completion of the saccadic task, participants were given a 3-min break to remove the VR glasses. Subsequently, the system as recalibrated before the anti-saccade task was initiated.

### 2.3 Symptom assessment

Hoehn and Yahr scale and Movement Disorder Society Unified Parkinson’s Disease Rating Scale III (MDS-UPDRS-III) (11) were used to assess motor symptoms of PD. Neuropsychological assessment was performed using MMSE, Montreal Cognitive Assessment (MoCA), Hamilton Depression Rating Scale (HAMD17) and Hamilton Anxiety Rating Scale (HAMA) to evaluate non-motor symptoms. Clinical motor subtypes (including tremor-dominance, akinetic-rigid and mixed subtypes), freezing of gait, and non-motor symptoms (orthostatic hypotension, bladder dysfunction, and sweating abnormalities) were assessed by two neurological specialists.

The MMSE consists of 5 items, including orientation, recall, memory, language, attention and calculation. The scores for MMSE range from 0 to 30, a score of <27 is considered to indicate cognitive impairment. MoCA contained 8 items, such as memory, attention, executive function, visuospatial skills, language skills and calculation, with a total score of 30. A score of ≥26 is considered to be normal. For subjects with 12 years or less education, one point was added to the total MoCA score. The HAMD-17 comprises 17 items (such as depressed mood, sleep disorders, appetite and weight), with a total score range of 0–52. A score of <7 is considered to be normal, and the higher the total score, the more severe the depression. The HAMA contains 14 items, with a total score range of 0–56, where a score of 0–6 indicates no/minimal anxiety. Higher scores indicate more severe anxiety symptoms.

### 2.4 Assessment of eye movements and changes in pupillary response

Eye movements and pupillary response of all subjects were monitored and analyzed using an eye-tracking system (EyeKnow; Beijing CAS-Ruiyi Information Technology Co., Ltd., Beijing, China, No. 20242070467). Before beginning the test, all subjects received a pre-test exercise to familiarize themselves with the testing procedure. During the test, a 1-min black screen interval was inserted between each set of tests for rest to minimize the effects of fatigue and distraction on the test results. And pupil changes were observed under a dark environment to keep the pupil diameter changing within a uniform range. Subjects wore a head-mounted eye tracking VR glasses that capture the changes in pupil size and record eye movement trajectories accurately. Before the eye movement test, each subject’s pupil position and gaze point were calibrated using a five-point calibration method. Subjects completed two types of saccade task, pro-saccade and anti-saccade tasks. In the pro-saccade task, subjects were required to look toward the new a stimulus quickly; conversely, in the anti-saccade task, subjects were required to look in the opposite direction of the stimulus when the stimulus appeared. The test began with subjects holding their gaze on a fixation stimulus for 1 s, then a new stimulus appeared on the lateral side for 1.2 s. Subjects were given a 2-s rest period. Twenty saccades were performed in each direction, and the direction was selected randomly. Saccadic eye movement parameters, such as saccade accuracy, velocity and error correction rate, were automatically calculated by the eye-tracking system. The pupillary response parameters included pupil diameter changes and frequency domain features. During eye movement task, pupil dilation and constriction patterns occurred repeatedly, wavy curve was produced to display the trajectories of changes in pupil diameter over time. The frequency domain analysis of pupillary responses was performed using Fourier transform to extract characteristics from pupil contraction during saccadic tasks. These frequency features were used to assess pupil sensitivity during the tasks, with low-frequency features indicating task-related pupil changes, such as cognitive stress, and high-frequency features reflecting subtle fluctuations in pupil constriction, which are sensitive to minute changes. All parameters were automatically recorded and calculated by the eye-tracking system. In order to ensure the accuracy of the statistical results, abnormal data due to blinking or measurement errors were excluded from the analysis.

### 2.5 Statistical analysis

Statistical analyses were performed using SPSS 23.0 statistical software. Continuous variables conforming to normal distribution was expressed as mean ± standard deviation (SD) and comparison between two groups was conducted by independent-sample *t*-test. Non-normally distributed continuous variable were expressed as median with interquartile range (Q1–Q3), and comparison between the two groups were performed using the Kruskal-Wallis rank sum test. Categorical variables were expressed as numbers or percentages (%) and analyzed using the chi-squared test. Shapiro–Wilk test was used to assess whether the features obeyed a Gaussian distribution. If they obeyed a Gaussian distribution, a parametric *t*-test was used for binary classification. Otherwise, a nonparametric Mann–Whitney U-test was used for binary classification. A *p*-value <0.05 was considered statistically significant.

## 3 Results

### 3.1 Comparison of general clinical data between the two groups

A total of 43 patients with early-stage PD in H&Y stages 1–2.5 were included in the study. There were 14 (32.6%) males and 29 (67.4%) females. The average H&Y stage was 2.1 ± 0.7. The average age of onset was 64.2 ± 8.4 years and the disease duration was 3.3 ± 1.8 years. In terms of PD motor subtypes, 14 (32.6%) patients had a tremor dominant subtype, 22 (51.2%) had an akinetic-rigid subtype, and 7 (16.3%) had a mixed type. And 3 (7.0%) patients reported the presence of freezing gait. The most common non-motor symptoms was bladder dysfunction (*n* = 16, 37.2%), followed by orthostatic hypotension (*n* = 4, 9.3%) and sweating abnormalities (*n* = 2, 4.7%). The mean HAMA score was 12.1 ± 5.2 points, the mean HAMD score was 12.2 ± 13.3 points, the mean MMSE score was 26.5 ± 2.3 points, and the MoCA-BC score was 23.6 ± 4.2 points.

A total of 25 healthy controls were included, with 14 (56.0%) males and a mean age of 62.3 ± 5.6 years. There were statistically significant differences in the average age between the two groups (*p* = 0.014, Table 1).

**Table 1:** Demographic and clinical characteristics of patients with early-stage Parkinson’s disease and healthy controls.

	PD group (*n* = 43)	Healthy control group (*n* = 25)	*t*/z/χ^2^	*p*
Age (year, mean ± SD)	64.2 ± 8.4	62.3 ± 5.6	−3.5776	0.0145
Male (*n,%*)	14 (32.6)	14 (56.0)	1.1277	0.28825
Age of onset (year, mean ± SD)	64.2 ± 8.4			
Disease duration (year, mean ± SD)	3.3 ± 1.8			
MDS-UPDRS-III score (point, mean ± SD)	24.9 ± 7.2			
Hoehn and Yarh stage(mean ± SD)	2.1 ± 0.7			
LEDD (mg/d, mean ± SD)	425.7 ± 193.3			
Motor subtype of PD (*n,%*)				
Tremor dominant subtype	14 (32.6)			
Akinetic-rigid subtype	22 (51.2)			
Mixed type	7 (16.3)			
Freezing of gait (*n,%*)	3 (7.0)			
Non-motor symptoms (*n,%*)				
Orthostatic hypotension	4 (9.3)			
Bladder dysfunction	16 (37.2)			
Sweating abnormalities	2 (4.7)			
HAMA score (point, mean ± SD)	12.1 ± 5.2			
HAMD score (point, mean ± SD)	12.2 ± 13.3			
MMSE score (point, mean ± SD)	26.5 ± 2.3			
MoCA-BC score (point, mean ± SD)	23.6 ± 4.2			

PD, Parkinson’s disease; LEDD, levodopa equivalent daily dose; MDS-UPDRS-III, Movement Disorder Society Unified Parkinson’s Disease Rating Scale III; HAMA, Hamilton Anxiety Rating Scale; HAMD, Hamilton Depression Rating Scale; MMSE, Mini-Mental State Examination; MoCA-BC, Montreal Cognitive Assessment.

### 3.2 Differences in saccadic eye movement parameters between the two groups

The pro-saccade accuracy was significantly lower (84.8% ± 24.6% vs. 93.3% ± 16.6%, *p* = 0.04), the average time taken to complete the pro-saccade was significantly prolonged (438.8 ± 133.1 ms vs. 377.5 ± 72.5 ms, *p* = 0.009), and the average pro-saccade velocity was significantly decreased in the PD group than in the healthy control group (Table 2). Furthermore, PD patients had significantly lower anti-saccade error correction rate than healthy controls (26.8 ± 31.2% vs. 45.1 ± 34.7%, *p* = 0.03, Table 2).

**Table 2:** Comparison of saccadic eye movement parameters between the two groups.

Eye movement parameters	PD group (*n* = 43)	Healthy control group (*n* = 25)	*t*	*p*
Pro-saccade accuracy (%)	84.8 ± 24.6	93.3 ± 16.6	398.0	0.04
Pro-saccade latency (ms)	269.5 ± 116.4	283.3 ± 100.3	525.5	0.88
Time taken to complete the pro-saccade (minimum, ms)	266.0 ± 85.4	252.8 ± 64.1	652.0	0.15
Time taken to complete the pro-saccade (average, ms)	438.8 ± 133.1	377.5 ± 72.5	741.5	0.01
Pro-saccade velocity (average, °/s)	206.5 ± 107.1	279.5 ± 105.6	−2.72	0.01
Pro-saccade velocity (maximum, °/s)	424.3 ± 197.9	536.5 ± 141.6	418.0	0.13
Anti-saccade accuracy (%)	42.5 ± 30.0	37.8 ± 25.0	595.5	0.46
Anti-saccade latency (ms)	300.8 ± 181.9	238.9 ± 140.1	654.0	0.14
Time to complete the anti- saccade (minimum, ms)	252.1 ± 175.8	202.1 ± 138.8	651.0	0.15
Time to complete anti-saccade (average, ms)	495.3 ± 158.8	437.8 ± 103.9	0.0	1.0
Anti-saccadeerror correction rate (%)	26.8 ± 31.8	45.1 ± 34.7	371.5	0.03
Time taken to correct anti-saccade errors (average, ms)	247.1 ± 236.8	310.0 ± 191.6	451.5	0.27
Anti-saccade velocity (average, °/s)	154.8 ± 81.3	184.5 ± 101.1	−1.33	0.19
Anti-saccade velocity (maximum, °/s)	386.3 ± 184.8	457.5 ± 243.4	452.5	0.28

### 3.3 The number of amplitudes of pupil contraction at different frequencies in PD patients

**Figure 1:**
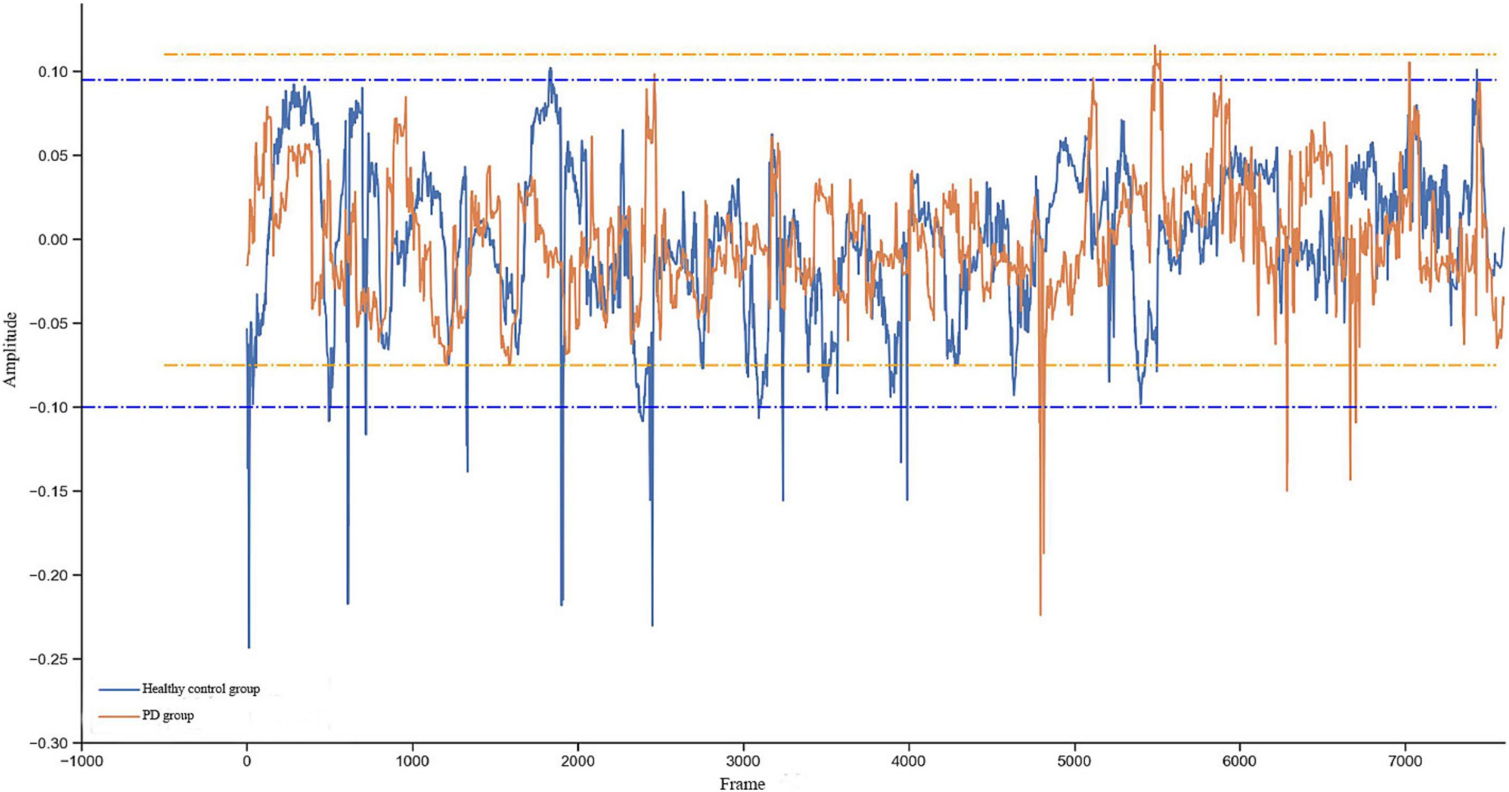
Differences in the trajectories of changes in pupil diameter between patients with early-stage Parkinson’s disease and healthy controls.

Significant differences were found in the trajectories of changes in pupil diameter between the two groups (Figure 1). After extraction of frequency-amplitude characteristic features of pupil contraction from the spectra of the eye movement signals of PD patients, it can be seen that regardless of which task was performed, the amplitudes of movement signals of both the left and right eyes at all frequencies during task were significant. The number of significant frequencies in both eyes at low (0–6 Hz), medium (7–12 Hz) and high frequencies (13–19 Hz) were 23, 9, and 16, respectively, for pro-saccades, 10, 29, and 43, respectively, for anti-saccades (Table 3).

**Table 3:** Number of significant amplitudes of pupil contraction at different frequency ranges in PD patients.

		Significant frequencies (*n*)		
		Low frequency (0–6 Hz)	Medium frequency (7–12 Hz)	High frequency (13–19 Hz)
Pro-saccades	Left eye	11	4	10
	Right eye	12	5	6
Anti-saccades	Left eye	4	11	18
	Right eye	6	18	25

### 3.4 Intergroup variations in pupillary response amplitude across frequency bands

**Figure 2:**
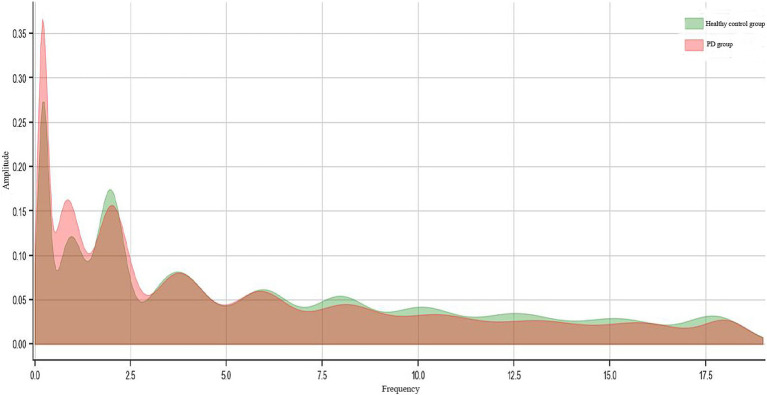
The average amplitude distribution of patients with early-stage Parkinson’s disease and healthy controls.

The average amplitude distribution of subjects in the two groups is shown in Figure 2 and it can be seen that there were significant differences in the amplitude of pupil contraction at different frequencies and the frequency at which the amplitude peak occurred between the PD and healthy control groups. The average frequency amplitude values across all frequency bands during saccade tasks differed significantly between the two groups. For example, during anti-saccade task, significant differences were found in the average amplitude values at 2.10 Hz (0.48 vs. 0.25), 11.80 Hz (0.10 vs. 0.17) and 17.40 Hz (0.07 vs. 0.12) between the two groups (Table 4).

**Table 4:** Comparison of average frequency amplitude values at different frequencies between the two groups.

		Low frequency (2.10 Hz)		Medium frequency (11.80 Hz)		High frequency (17.40 Hz)	
		Healthy control group	PD group	Healthy control group	PD group	Healthy control group	PD group
Pro-saccades	Left eye	0.3982	0.4659	0.1048	0.1333	0.0939	0.0810
	Right eye	0.2700*	0.5152*	0.0866	0.1142	0.0866	0.1003
Anti-saccades	Left eye	0.4341	0.4563	0.1412*	0.0949*	0.1416*	0.0787*
	Right eye	0.2548*	0.4786*	0.1660*	0.1017*	0.1198*	0.0664*

^*^Significant difference (*p* < 0.05).

### 3.5 Development of machine learning diagnostic model

After selection of significant features, Random Forests method was used for classification task, machine learning diagnostic models were established to differentiate between PD patients and healthy controls. Performance evaluation results showed that the diagnostic model combining basic eye-movement features and frequency features of pro-saccades and anti-saccades achieved an accuracy of up to 79% in differentiating early-stage PD patients from healthy controls (Table 5).

**Table 5:** Performance evaluation metrics for machine learning diagnostic modes.

Model	Accuracy	Precision	Recall	F1 score
Basic eye movement features	0.6306	0.6331	0.6148	0.5907
Frequency features of pro-saccades	0.7527	0.7369	0.7248	0.6638
Frequency features of anti-saccades	0.7956*	0.7924	0.7503	0.7550*
Frequency features of pro-saccades + basic eye movement features	0.7802	0.7076	0.6796	0.6681
Frequency features of anti-saccades + basic eye movement features	0.7875	0.7813	0.7575*	0.7478
All features	0.7908	0.8239*	0.7512	0.7415

^*^Significant difference (*p* < 0.05).

## 4 Discussion

This is a cross-sectional study exploring the characteristics of eye movements and pupillary response in early-stage PD patients. Unlike previous studies, we used a combination of saccadic eye movement tasks and virtual reality visualization framework to induce eye movements and monitor characteristic pupillary response, and adopted eye movement and pupillary response abnormalities observed to assist in the clinical diagnosis of early-stage PD. Our findings revealed prolonged time taken to complete the pro-saccade, reduced average pro-saccade velocity, decreased pro-saccade accuracy, as well as decreased anti-saccade error correction rate in early-stage PD patients. Our findings also showed characteristic abnormalities of pupil constriction in early-stage PD patients.

The study had a higher percentage of female participants (67.4%) in the early-stage PD group compared to the healthy control group (56.0% males), which contrasts with the typical male predominance in PD (male-to-female ratio ~1.4:1) (12). This imbalance may stem from single-center, regional, or demographic factors, referral bias, or differences in disease presentation. While we adjusted for sex in our analyses, the imbalance could affect the generalizability of our findings. Future studies with balanced sex distributions or sex-stratified analyses are needed to explore potential sex-specific influences and clarify the reasons for this unusual distribution.

Previous anatomical and neuroimaging findings have suggested that the majority of eye movement alterations may be associated with damage to specific areas of the brain that involved in the control of eye movements, like brainstem, basal ganglia region and cerebellum. Therefore, eye movement abnormalities can be used as an objective method to quantify related diseases (13). A growing number of studies have reported that eye movement abnormalities may manifest in the early stages (14) or even the prodromal stages of PD (15), thus valid identification of eye movement abnormalities is helpful for early diagnosis of PD and assessment of disease progression. Eye movements have emerged as a potential objective biomarker for assessing characteristic movement disorders and cognitive impairment (16–18). However, to date, there is a lack of sufficient reliable evidence to diagnose and differentiate PD based on ocular symptoms and signs. In this study, we found that patients with early PD had a prolonged time taken to complete the pro-saccade and a reduced average pro-saccade velocity. These results are consistent with the findings of Painous et al. (19) It is currently believed that pathway for saccades is composed of the cerebral cortex, subcortical structures and brainstem. Saccades can be facilitated and inhibited by projections to the superior colliculus (SC) (20). The frontal and parietal cortices regulate superior colliculus (SC) activity through dual pathways: a direct excitatory glutamatergic connection and an indirect basal ganglia pathway via the substantia nigra pars reticulata (SNr). The SNr exerts tonic GABAergic inhibition on the SC to suppress saccadic eye movements. This inhibitory control is modulated by three distinct pathways implicated in Parkinson’s disease (PD): (1) the direct striatonigral pathway (caudate→SNr) disinhibits saccades; (2) the indirect striatopallidal pathway (GPe → STN → SNr) enhances saccade suppression; and (3) the hyperdirect pathway (cortex→STN → SNr) provides rapid saccade cancelation through excitatory subthalamic input. Taken together, this system forms an inhibitory pathway to the SC, that may also be involved in reward-modulated and motivated behavior (21, 22), and plays a decisive role in the initiation of saccades (23, 24). It is hypothesized that the presence of saccadic abnormalities in PD patients may be related to failed removal of SNr-induced tonic inhibition on SC due to a reduction in dopamine in the basal ganglia (25). Stefsnescu et al. (26) found that saccadic abnormalities were commonly seen in PD patients, voluntary saccades were typically generated in the initial phase, which were less accurate and hypometric. And with disease progression, saccade latency was increased and velocity was decreased (27), with the presence of visually guided saccades. In line with the previous findings, our study showed that in the pro-saccade task, time taken to complete the saccade was prolonged, with decreased velocity and reduced accuracy in early-stage PD patients.

The results of this study also showed reduced anti-saccade error correction rate in early-stage PD patients when compared to healthy controls. There are different types of saccadic eye movements, visually-guided pro-saccades and anti-saccades. Pro-saccades are conscious, reflexive saccades in response to the object of interest. Anti-saccades are complex eye movements. During an anti-saccade task, participants are required to maintain their gaze on a visual stimulus in the center of the screen. After a fixation interval, the visual stimulus appears randomly on either side of the screen, and participants are instructed to make a saccade to the location opposite the appearance of the stimulus. A previous study reported that early-stage PD patients were primarily impaired in the performance of complex saccades (i.e., anti-saccades), and the degree of impairment increased gradually with disease progression, manifesting by decreased accuracy and prolonged latency of saccades (28). In line with the previous findings, our study showed that in comparison with healthy controls, early-stage PD patients exhibited a decreased ability to correct anti-saccade errors, while no significant differences were found in the latency and velocity of anti-saccades between the two groups. However, previous study also reported abnormal anti-saccade task performance in other neurodegenerative diseases (29).

During the eye movement task, repetitive patterns of pupil dilation and constriction occur. The repetitive behavior resembles wavy curve of pupil diameter changes, which can be used for signal processing in the frequency domain, thus extracting features from the signals. Previous studies adopted eye movement tracking devices such as infrared eye-tracker (30) and head-mounted eye-tracker based on PC technology (31) to measure pupil changes in PD patients. In this study, we designed a VR-based system to reveal the characteristic manifestations of pupil constriction in early-stage PD patients. The horizontal lines of different colors depicted represent the maximum and minimum amplitude ranges for each subject, with corresponding low-frequency amplitudes. Small fluctuations during saccades reflect high-frequency amplitudes, showing small fluctuations in pupil constriction. Low-frequency features represent task-related pupil changes, including pupil changes caused by changes in the cognitive stress imposed on an individual, while high-frequency features capture subtle fluctuations and reflect pupil sensitivity (32). Low- and high- frequency features both differed significantly between PD patients and healthy controls. In addition, the average amplitude distribution graph drew based on the frequency features, i.e., the difference in amplitude at different frequencies between different populations, can distinguish between diseased and healthy populations. Our study similarly showed differences in frequency features between the two groups.

In contrast to traditional eye movement parameters such as fixation duration, saccade velocity and latency, frequency-domain analysis provides a unique perspective. In the frequency domain, eye movement signals can be converted into spectrum by Fourier transform, revealing amplitude and phase information associated with different frequency components (33). It is worth noting that frequency, as a variable in field of real numbers, has a range of values that are infinite. Even a cutoff frequency is set in the analysis to filter out noise or irrelevant high-frequency components, rich frequency amplitude features can still be extracted from the spectrum. These frequency features reflect subtle changes in eye movements, with more significant features being provided when compared to eye movement features, which are more conducive to identify early-stage PD patients (34). In the present study, we found that regardless of pro-saccade task or anti-saccade task, the amplitudes of movement signals of both the left and right eyes at all frequencies during task were significant in PD patients. In addition, the number of significant frequencies at different frequency range varied for different tasks. Further analysis revealed that the average frequency amplitude values across all frequency bands during saccade tasks also differed significantly between the two groups. For example, during anti-saccade task, significant differences were found in the average amplitude values for the right eye at 2.10 Hz (0.48 vs. 0.25), 11.80 Hz (0.10 vs. 0.17) and 17.40 Hz (0.07 vs. 0.12) between the two groups.

From these above-mentioned results, there are several observations that deserve our attention. First, regardless of which task was performed, several significant amplitudes were found in different frequency bands, which cover different frequency ranges, providing more valid features when compared to traditional eye movement parameters. Second, for pro-saccades, significant frequencies were mostly found at a low frequency range; conversely, for anti-saccades, significant frequencies were mostly found at a high frequency rang. Third, anti-saccades yielded more significant frequencies, likely due to the high cognitive demand of the task, which may induce irregular pupil constriction and help identify early-stage PD. However, factors such as stress, cognitive impairment (evidenced by low MoCA-BC scores of 23.6 ± 4.2 in the study), and impulsivity in PD patients could influence results. Stress from the VR system may affect pupillary responses and eye movement accuracy due to its impact on autonomic nervous system activity while cognitive deficits and impulsivity could impair task performance, particularly in pro-saccades and anti-saccade tasks. While VR-based eye-tracking shows promise for early PD diagnosis, the influence of stress, cognition, and impulsivity should be acknowledged. Addressing these factors in future studies will enhance the reliability and clinical applicability of VR-based eye-tracking as a diagnostic tool for early PD.

In this study, Random Forest was used for classification task. Different metrics including accuracy, precision, recall and F1 score are used to evaluate the performance of the classification model. In the binary classification saccade task, the model with all of the features achieved an accuracy of up to 79%, whereas using basic eye movement features (including pro-saccade, anti-saccade and related parameters) achieved an accuracy of 63%. Therefore, models combining basic eye-movement features and specific frequency features of saccades typically showed good performance compared to the models using these features alone. Furthermore, frequency features of saccades produce more accurate results for discrimination of PD patients from healthy controls compared to basic eye-movement features.

The study has some limitations. First, this is a single-center study with a small sample size. It is necessary to further expand the sample size and conduct multicenter study in future to confirm the importance of ocular symptoms and signs in the progression of PD. Second, a previous autopsy study documented an accuracy of 80% for the clinical diagnosis of PD at early stages (35). In this study, the diagnosis of early-stage PD mostly relies on clinical experience of clinicians, there may be potential for misdiagnosis. Therefore, close follow-up of patients and differentiation between PD and parkinsonism-plus syndromes should be considered to ensure diagnostic accuracy. Furthermore, in this study, patients who took dopaminergic medications were not excluded. Drug-induced eye movement abnormalities should be taken into account. Further studies are needed to compare the differences in eye movements between early-stage PD patients who took medicine and those who did not.

## 5 Conclusion

In summary, our findings revealed saccadic eye movement abnormalities in early-stage PD patients through VR technology, including prolonged time taken to complete the pro-saccade, decreased velocity and accuracy of pro-saccades, and decreased anti-saccade error correction rate. Our findings also showed characteristic abnormalities of pupil constriction in early-stage PD patients. The diagnostic model combining basic eye-movement features and specific frequency features of saccades helps in improving diagnostic accuracy of early-stage PD. This study presents a non-invasive approach toward the diagnosis of early-stage PD with VR technology. The presence of abnormal eye movement and pupillary response measured using VR may be used as effective biomarkers for the diagnosis of early-stage PD.

## Data Availability

The original contributions presented in the study are included in the article/supplementary material, further inquiries can be directed to the corresponding author.
